# Glioma-Associated Sialoglycans Drive the Immune Suppressive Phenotype and Function of Myeloid Cells

**DOI:** 10.3390/pharmaceutics16070953

**Published:** 2024-07-19

**Authors:** Lenneke A. M. Cornelissen, Kim C. M. Santegoets, Esther D. Kers-Rebel, Sandra A. J. F. H. Bossmann, Mark Ter Laan, Daniel Granado, Gosse J. Adema

**Affiliations:** 1 Radiotherapy and OncoImmunology Laboratory, Department of Radiation Oncology, Radboud University Medical Center, 6525 GA Nijmegen, The Netherlands; 2 Department of Neurosurgery, Radboud University Medical Center, 6525 GA Nijmegen, The Netherlands

**Keywords:** glioma, sialoglycans, Siglecs, myeloid cells

## Abstract

The tumor microenvironment of glioblastoma IDH-wildtype is highly immune suppressive and is characterized by a strong component of myeloid-derived suppressor cells (MDSCs). To interfere with the immune suppressive functions of MDSCs, a comprehensive understanding on how MDSCs acquire their suppressive phenotype is essential. Previously, we and others have shown a distinct Sialic acid-binding immunoglobulin-like lectin (Siglec) receptor expression profile for MDSCs in glioblastoma. Siglec receptors can transmit inhibitory signals comparable to PD-1 and are suggested to act as glyco-immune checkpoints. Here, we investigated how glioma specific Siglec-sialic acid interactions influence myeloid immune suppressive functions. Co-culturing monocytes with glioblastoma cells induced CD163 expression on the monocytes. Upon desialylation of the glioblastoma cells, this induction of CD163 was hampered, and furthermore, the monocytes were now able to secrete higher amounts of IL-6 and TNFα compared to fully sialylated glioblastoma cells. Additionally, Siglec-specific triggering using anti-Siglec-7 or Siglec-9 antibodies displayed a decreased TNFα secretion by the monocytes, validating the role of the Siglec–Sialic axis in the co-culture experiments. Together, our results demonstrate that glioblastoma cells induce a myeloid immune-suppressive phenotype that could be partly rescued by lowering the glioblastoma-associated sialic acid levels. This manuscript supports further research of the Siglec–Sialic acid axis in the context of glioblastoma and its potential to improve clinical outcome.

## 1. Introduction

Cancer immunotherapy has firmly been established as the fourth pillar of cancer care. Many tumors, however, evolve mechanisms to create and sustain an immune-suppressive tumor microenvironment (TME) that hampers cancer immunotherapy treatment outcome. Diffuse glioma is a heterogeneous group of brain tumors with a diverse set of molecular backgrounds. Glioblastoma IDH-wildtype is the most aggressive and by far the most frequent form of diffuse glioma, and is difficult to treat due to the invasive growth pattern, extensive hypoxic areas, and immune-suppressive TME. Early laboratory studies [1] and clinical immunotherapy trials are ongoing, but the overall response rates remain low for glioblastoma [2,3]. The glioblastoma TME is characterized with a strong myeloid compartment, including myeloid-derived suppressor cells (MDSCs) and tumor-associated macrophages [4,5]. MDSCs hamper immune cell functions [6], and thereby contribute to an immune suppressive TME. A better understanding of how MDSCs acquire their immune-suppressive phenotype could provide new targets to interfere with, and thereby improve, cancer immunotherapy efficacy in glioblastoma patients.

MDSCs express sialic acid-binding immunoglobulin-like lectin 3 (Siglec-3 or CD33). Siglec-3 is a member of the Siglec receptor family that are broadly expressed on immune cells and are well-known for their immune-modulatory functions [7,8,9]. For instance, the triggering of Siglec-3 in MDSCs has been shown to promote expansion of MDSCs, and it induces the secretion of immunosuppressive cytokines by immature myeloid cells [10]. At their intracellular part, the majority of Siglecs have a tyrosine-based signaling inhibitory motif (ITIM) very similar to the immune checkpoint receptor PD-1. Siglecs are carbohydrate-binding receptors that recognize sialoglycans, a sialic acid monosaccharide-carrying glycan conjugated to proteins or lipids. The TME displays enhanced expression of sialoglycans, independent of the tumor type, that have been associated with poor prognosis [11,12]. Due to their prominent location on the cell surface, sialic acids mediate and modulate complex cellular and cell–protein interactions, including those between tumors and immune cells [13,14]. Together, it has been postulated that tumor cells exploit the Siglec–sialic acid axis to evade immunity [7,13,15].

Previously, we assessed the Siglec expression profile on MDSCs from glioma patients, and the Siglec ligand expression on freshly isolated glioma cells. Glioma-infiltrating MDSCs express Siglec-3, -5, -7, and -9, whereas glioma cells predominantly express sialoglycans recognized by Siglec-7 and Siglec-9 [16]. Previously, the role of Siglec in preclinical glioblastoma models and its impact on the anti-tumor immune response has been demonstrated [17]. However, to what extent Siglec–sialic acid interactions influence MDSC function in humans remains largely unknown. Here, we demonstrate that glioma cells induce an immune suppressive phenotype and function of myeloid cells, which can be partially reversed by disrupting the Siglec–sialic acid axis. Together, the data suggest that the Siglec–sialic acid axis could be targeted to interfere with the immune-suppressive TME to ultimately improve cancer immunotherapy efficacy in glioma patients.

## 2. Materials and Methods

### 2.1. Glioma Cell Culture and Sialic Acid Inhibitor Treatment

Human glioblastoma cell line T98G (CRL-1690), obtained from the American Type Culture Collection (Manassas, VA, USA), was grown in Dulbecco’s Modified Eagle’s Medium (DMEM) (Gibco, New York, NY, USA) with GlutaMAX (Gibco, New York, NY, USA) supplemented with 10% heat-inactivated FBS (Greiner Bio-one, Fricken-hausen, Germany) and 100 U/mL Penicillin–Streptomycin (Gibco, New York, NY, USA). The cells were regularly screened for mycoplasma contamination (Lonza, Walkersville, MD, USA) and incubated in a humidified CO_2_ incubator at 37 °C. The sialic acid inhibitors Ac_5_3F_ax_Neu5Ac and SiaFEt were kindly provided by dr T. Boltje, and were synthesized as described previously [18,19,20]. Ac_5_3F_ax_Neu5Ac, SiaFEt, or DMSO control were added to the T98G culture for 4 days. Cells were intensively washed and put back in the culture for the recovery assay or were directly used to start the co-culture experiments or to perform flow cytometry analysis.

### 2.2. Monocyte Isolation and Co-Culture Experiments

Buffy coats (Sanquin, Nijmegen, The Netherlands) and/or peripheral blood samples were collected from healthy individuals and glioma patients undergoing neurosurgical resection or biopsy for intracranial tumors at the Radboud University Medical Center (Radboudumc, Nijmegen, The Netherlands). All patients had histologically proven brain tumors diagnosed by neuropathologists of the Radboudumc. The tumors were classified according to the WHO 2016 Classification of tumors of the Central Nervous System [21], and encompassed three glioblastoma’s, IDH-wildtype, and one diffuse astrocytoma, IDH-wildtype (WHO grade II). Immediately after the blood samples were obtained, processing of these samples was started. Peripheral blood mononuclear cells (PBMCs) were isolated using a Lymphoprep density gradient. PBMC fraction was subsequently used to purify CD14^+^ monocytes using MACS CD14-positive selection Microbeads (Miltenyi Biotec, Leiden, The Netherlands)) following the manufacturer’s protocol.

At the start of the co-culture, T98G cells and CD14^+^ monocytes were simultaneously added to a 96-U-bottom well, and were cultured in RPMI medium (Gibco, New York, NY, USA) supplemented with 10% heat-inactivated FCS, 1% Penicillin–Streptomycin, and 1% Ultra-glut (Lonza, BEBP17-605E). To investigate effects on cytokine production and secretion, the co-culture was stimulated with 100 ng/mL Lipopolysaccharide at day 4 overnight. The co-culture experiments were incubated in a humidified CO_2_ incubator at 37 °C.

### 2.3. Siglec Activation in Monocytes

Monocytes were isolated from buffy coats as described above. The monocytes were plated out overnight in RPMI medium (Gibco, New York, NY, USA) supplemented with 10% heat-inactivated FCS, 1% Penicillin–Streptomycin, 1% Ultra-glut (Lonza, BEBP17-605E), and 50 ng/mL M-CSF (Preprotech, London, UK). Subsequently, the medium was replaced with fresh medium containing 10 ng/mL Lipopolysaccharide and either 5 µg/mL anti-Siglec-7 monoclonal antibody (Biolegend, Amsterdam, The Netherlands), 5 µg/mL anti-Siglec-9 monoclonal antibody (R&D systems, Minneapolis, MN, USA), or 5 µg/mL isotype antibody (Bio X Cell, Lebanon, PA, USA). After 16–20 h, supernatant was collected and used for cytokine ELISA.

### 2.4. Flow Cytometry and Cytokine ELISA

Using standardized flow cytometry protocols as described previously [16], cells were stained for different viability dyes, antibody membrane markers, and lectins. In short, the cells were washed with PBS and stained with eFluor780 viability dye. For staining with biotinylated lectin, the cells were washed with carbo-free blocking solution and incubated with MALII (5 µg/mL) or SNA (1 µg/mL) to detect α2-3 and α2-6 sialic acids, respectively (both Vector Laboratories, Newark, NJ, USA), for 45 min at 4 °C. Next, the cells were washed with PBA (PBS with 1% bovine serum albumin and 0.02% sodium azide) and incubated with Streptavidin-PE (eBioscience, San Diego, CA, USA) for 15 min at 4 °C. For staining with antibody membrane markers, cells were washed with PBA and incubated with anti-CD45-PE (clone HI30, Biolegend, Amsterdam, The Netherlands), HLA-DR-BV421 (clone G46-6, BD Bioscience, Franklin Lakes, NJ, USA), CD206-Alexa Fluor 488 (clone 19.2, eBioscience, San Diego, CA, USA), CD163-APC (clone eBioGHI/61, eBioscience, San Diego, USA), CD86-PE-Cy7 (clone 2331 (FUN-1), BD Bioscience, Franklin Lakes, NJ, USA), and Siglec-1-Alexa Fluor 647 (clone 7-239, Biolegend, Amsterdam, The Netherlands) for 20 min at 4 °C. Cells were washed and acquired on CytoFlex LX (Beckman Coulter, Brea, CA, USA) or BD FACSCanto (BD Bioscience, Franklin Lakes, NJ, USA), and were analyzed using FlowJo v10 software (Tree Star, Ashland, OR, USA).

Cytokine levels in the supernatant were quantified using sandwich ELISAs for IL-6, TNFα, and IL-10 (eBioscience, San Diego, CA, USA) according to the manufacturer’s instructions.

### 2.5. Statistical Analysis

Statistical analysis was performed using GraphPad Prism 10.1.2. For multiple comparisons, a one-way ANOVA test, and between two groups, a paired *t*-test, was performed (* *p* < 0.05, ** *p* < 0.01, *** *p* < 0.001; ns, not significant).

## 3. Results

### 3.1. Sialic Acid Inhibition Results in Long-Lasting Reduction in Sialylation in the Glioblastoma T98G Cell Line

**Figure 1:**
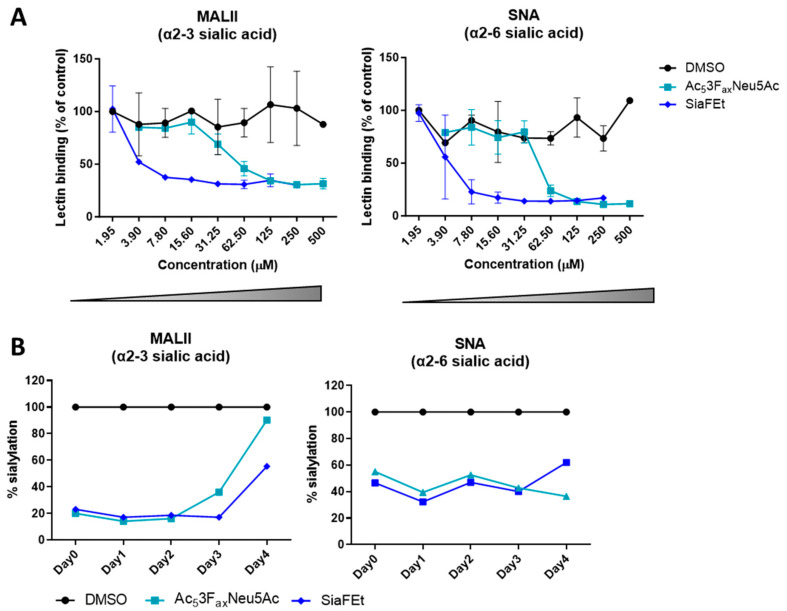
Sialic acid inhibitors stably reduce the sialoglycan content in the human glioblastoma cell line T98G. (**A**) The two sialic acid inhibitors Ac_5_3F_ax_Neu5Ac and SiaFEt tested cause a dose-dependent inhibition of sialic acid expression on T98G cells compared to DMSO control. T98G cells were treated for 4 days, and expression of α2-3- and α2-6-linked sialic acid was analyzed using MALII and SNA lectins, respectively. The data presents *n* = 2 independent experiments. (**B**) T98G cells were treated with either 1000 µM Ac_5_3F_ax_Neu5Ac or 125 µM SiaFEt. After 4 days, the inhibitors were extensively washed away. The T98G cells display a reduction is sialic acid expression for up to 4 days.

To explore the sialic acid-mediated impact of glioblastoma cells on myeloid functions, we established a glioblastoma cell model expressing high and reduced levels of sialic acids. Previously, we reported that the glioblastoma cell line T98G expresses high levels of sialoglycans [16]. Here, we continued using T98G cells and tested its sensitivity for two different sialic acid inhibitors (Sia ↓), namely Ac_5_3F_ax_Neu5Ac and C-5 carbamate-based inhibitor (SiaFEt) [20]. Both sialic acid inhibitors showed a dose dependent decrease on T98G sialylation measured with lectins specific for either α2-3- or α2-6-linked sialic acids (Figure 1A). Compared to the DMSO control, SiaFEt was found to be more potent in reducing T98G sialylation than Ac_5_3F_ax_Neu5Ac. Where SiaFEt achieves a 50% sialic acid reduction at 3.9–7.8 µM, Ac_5_3F_ax_Neu5Ac needs 31.25–62.50 µM to achieve similar sialic acid reduction. For subsequent experiments, we applied 125 µM SiaFEt or 1000 µM Ac_5_3F_ax_Neu5Ac to generate T98G cells with reduced sialic acid expression. Next, we analyzed the recovery time of T98G sialylation upon sialic acid inhibitor treatment by extensively washing away the inhibitors. The effect of the sialic acid inhibitors remained present up to 4 days (Figure 1B).

Together, the human glioblastoma T98G cell line was shown to be sensitive for sialic acid inhibitor treatment, resulting in a reduction in sialylation up to 4 days.

### 3.2. Glioblastoma Cells Induce Sialic Acid Dependent Immune Suppressive Phenotypes in Monocytes

**Figure 2:**
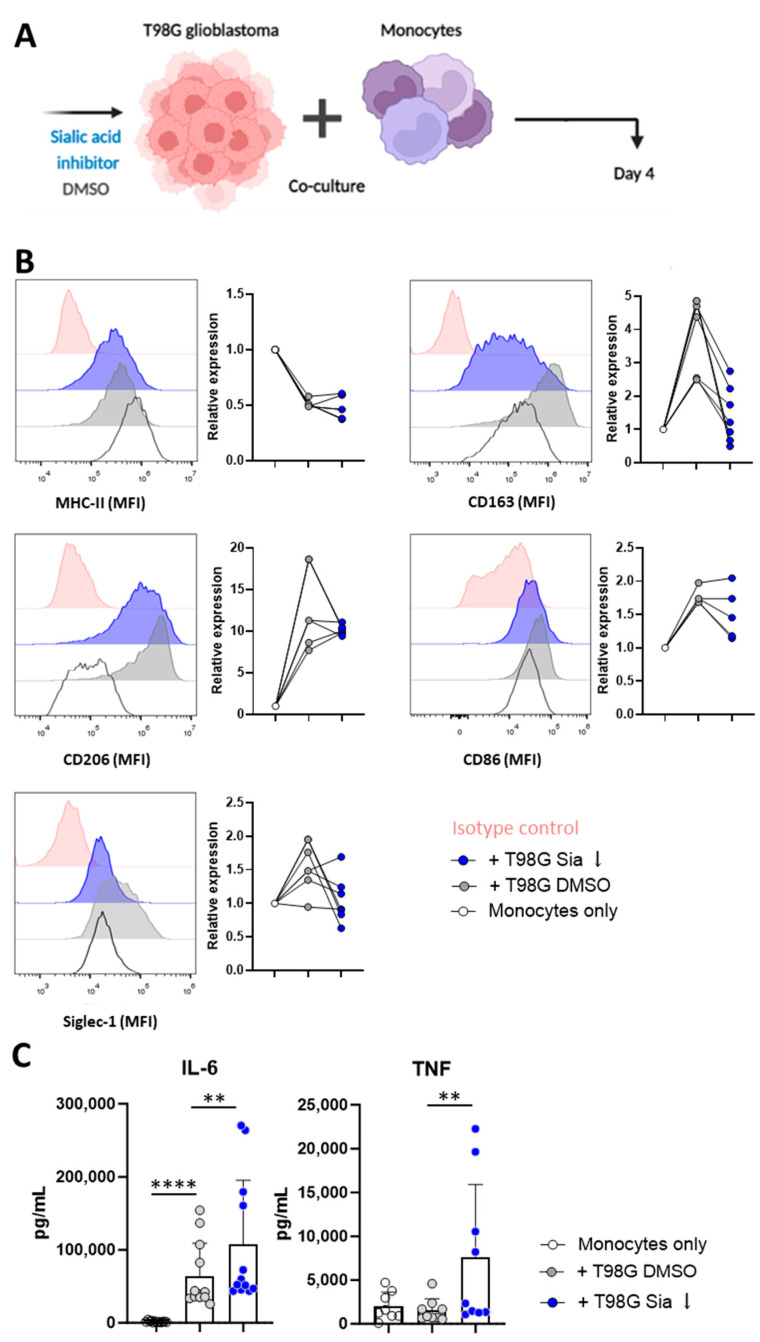
Sialic acids on T98G glioblastoma cells drive immune-suppressive phenotypes in monocytes. T98G cells were pretreated with sialic acid inhibitors or DMSO controls, and were subsequently co-cultured with monocytes for 4 days. (**A**,**B**) Flow cytometry analysis of MHC-II, CD163, CD206, CD86, and Siglec-1 after 4 days of co-culture. Each dot represents one biological replicate. MFI; mean fluorescence intensity. (**C**) After 4 days, cells were stimulated with 100 ng/mL LPS overnight. Supernatant was collected and cytokine secretion was analyzed. Each dot represents one of the duplo or triplo measurements of one biological replicate. A one-way ANOVA test was performed (** *p* < 0.01, **** *p* < 0.0001).

As a next step, we investigated the influence of glioblastoma T98G cells on monocytic functions and the role of T98G-associated sialylation on this. Monocytes isolated (CD14+ selection) from healthy control buffy coats were co-cultured with T98G cells treated with either the sialic acid inhibitor (T98G Sia ↓) or DMSO control (T98G DMSO) (Figure 2A). After 4 days of co-culturing with T98G cells, monocytes displayed phenotypical characteristics of tumor associated myeloid cells characterized by a 50% reduction in MHC-II MFI levels and high upregulation of the CD163 and CD206 marker compared to monocytes cultured without T98G cells (Figure 2B). Myeloid cell maturation was further confirmed with an increased CD86 expression upon T98G co-culture (Figure 2B). Siglec-1 is an endocytic receptor, and is exclusively expressed on monocytes and macrophages that have dual anti-tumor functions depending on the tumor type [22,23,24]. Siglec-1 was shown to be upregulated on the monocytes after T98G co-culture (Figure 2B). Interestingly, the upregulation of CD163 was abolished upon co-culture, with T98G cells being inhibited in their sialic acid expression.

As a next step, the tumor co-cultured monocytes were stimulated with 100 ng/mL lipopolysaccharide (LPS) overnight. Upon LPS treatment, the monocytes co-cultured with T98G Sia ↓ were able to secrete higher amounts of IL-6 and TNFα cytokines when compared to monocytes co-cultured with T98G DMSO (Figure 2C).

Together, the data suggest that T98G-monocyte interactions result in differentiation of tumor-associated myeloid cells with high expression of CD163 and hampered potential to secrete cytokines, which could partly be rescued by the removal of sialic acid expression on the T98G cells.

### 3.3. Glioblastoma Cells Induce Sialic Acid Dependent Immune Suppressive Phenotypes in Glioma Patient Derived Monocytes

**Figure 3:**
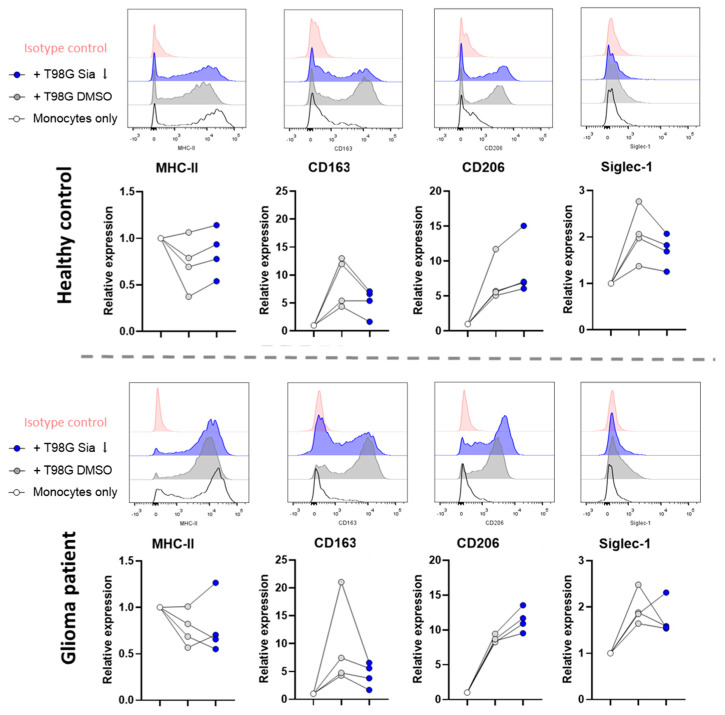
Sialic acids on T98G glioblastoma cells drive immune-suppressive phenotypes in glioma patient-derived monocytes. T98G cells were pretreated with sialic acid inhibitors or DMSO controls, and were subsequently co-cultured with monocytes obtained from healthy controls or glioma patients in parallel. After 4 days, MHC-II, CD163, CD206, and Siglec-1 expression were analyzed using flow cytometry. Each dot represents one biological replicate.

Next, we tested how T98G cells influence the functions of glioma patient-derived monocytes. Blood from glioma patients was drawn at the start of surgery and in parallel from a healthy volunteer. By drawing blood from a healthy volunteer instead of using buffy coats, the blood procedures were kept equal between glioma patient (PT) and healthy controls (HC). After 4 days of co-culture with either T98G Sia ↓ or T98G DMSO, HC-derived monocytes showed similar phenotypic characteristics as those previously observed with HC monocytes isolated from buffy coats (Figure 2B and Figure 3). Furthermore, the sialylation status of T98G Sia ↓ cells remained lower compared to T98G DMSO cells at the end of the co-culture experiment (Supplementary Figure S1).

**Figure 4:**
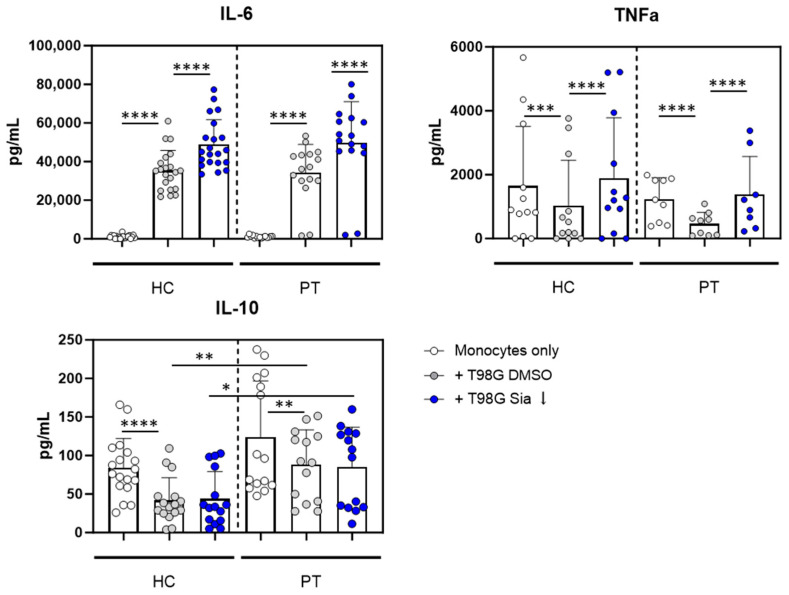
Sialic acids on T98G glioblastoma cells change cytokine secretion profiles in glioma patient and healthy control derived monocytes. T98G cells were pretreated with sialic acid inhibitors or DMSO control, and were subsequently co-cultured with monocytes obtained from healthy controls (HC) or glioma patients (PT) in parallel. After 4 days, cells were stimulated with 100 ng/mL LPS overnight. Supernatant was collected and cytokine secretion was analyzed. Each dot represents one of the duplo or triplo measurements of one biological replicate. A one-way ANOVA test was performed (* *p* < 0.05, ** *p* < 0.01, *** *p* < 0.001; **** *p* < 0.0001).

The T98G co-culture downregulated MHC-II and increased CD163, CD206, and Siglec-1 expression on both HC and PT monocytes. From these markers, again CD163 upregulation on HC and PT monocytes were shown to be dependent on T98G sialylation (Figure 3). Monocyte activation was confirmed by increased IL-6 and TNFα cytokine secretion upon T98G co-culture compared to monocytes only (Figure 4). Interestingly, like HC monocytes, PT monocytes were able to secrete higher amounts of IL-6 and TNFα cytokines upon sialic acid inhibitor treatment of the T98G cells compared to DMSO control. To assess the anti-inflammatory status of the monocytes, IL-10 secretion was analyzed. For both HC and PT monocytes, IL-10 was reduced upon T98G co-culture independent of the sialyation status. However, IL-10 levels were significantly higher in PT derived monocytes upon T98G co-culture compared to HC monocytes, validating the immune-suppressive onset of PT-derived monocytes (Figure 4).

Altogether, our data show that both HC and PT monocytes display immune-suppressive functions after interactions with the human glioblastoma T98G cell line. Sialic acid expression on T98G cells was found to be responsible at least partly for these immune-suppressive phenotypes.

### 3.4. Siglec Activation Validates the Involvement of Siglec Receptors in the T98G-Induced Immunosuppressive Myeloid Phenotype

**Figure 5:**
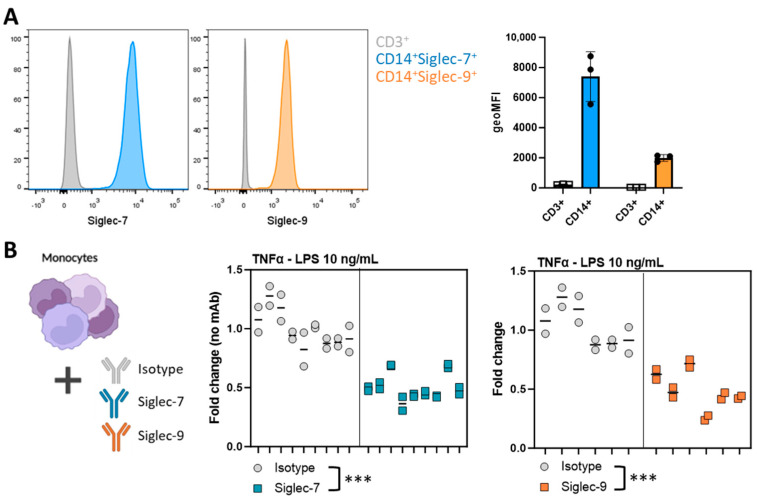
Siglec triggering of monocytes results in reduced TNFα secretion. (**A**) Monocytes (CD14^+^ PBMCs) express both Siglec-7 and Siglec-9, which is not present on CD3^+^ PBMCs. Each dot represents one biological replicate. (**B**) Monocytes were stimulated with 10 ng/mL LPS in combination with 5 µg/mL anti-Siglec-7, 5 µg/mL anti-Siglec-9, or 5 µg/mL isotype monoclonal antibodies (mAb). Both Siglec-7 and Siglec-9 mAb stimulation hampered TNFα secretion upon stimulation with LPS. Data are presented as Nested data of *n* = 9 (Siglec-7) or *n* = 6 (Siglec-9) biological replicates, with each performed in duplo. A paired *t*-test was performed. (*** *p* < 0.001).

As a next step, we investigated to what extent these observed effects were mediated by Siglec activation. Hereto, we stimulated healthy control monocytes that do express Siglec-7 and Siglec-9 (Figure 5A) with LPS in combination with anti-Siglec-7 or anti-Siglec-9 monoclonal antibodies (mAbs). Upon activation of both Siglec-7 and Siglec-9 receptors, monocytes decreased their TNFα secretion compared to isotype control (Figure 5B). In conclusion, Siglec-specific triggering using anti-Siglec-7 or Siglec-9 antibodies confirm the involvement of the Siglec receptors in the T98G-induced immunosuppressive myeloid phenotype.

## 4. Discussion

Sialoglycans present on cancer cells have been associated with the acquisition of multiple cancer hallmarks, including immune evasion. The functional properties of glioma-associated sialylation on myeloid cell function are not fully understood. Here, we investigated the contribution of glioma-specific sialylation on the induction of an immune suppressive phenotype in healthy controls and glioma patient-derived monocytes.

The glioma TME is enriched with MDSCs that are known to promote tumor progression [25,26,27,28]. Turning the immune suppressive TME into an immune permissive TME could unleash the immune system’s potential to control the tumor mass and improve immunotherapeutic outcomes. Repolarizing tumor-associated myeloid cells into cells contributing to tumor control is one of the current challenges to make the TME more immune permissive. We and others have shown that these glioma-infiltrating myeloid cells express multiple Siglec receptors [16] that recognize and signal upon binding with sialoglycans. Since Siglecs are well described for their immune suppressive modulatory capacities, the Siglec–sialic acid axis is an interesting target to reprogram the immune-suppressive glioma TME. Here, we have shown that indeed glioma cells are capable to induce an immune-suppressive phenotype in monocytes isolated from healthy control or glioma patient-derived blood. One of the markers used to classify monocytes/macrophages as tumor-supportive is the scavenger receptor CD163 [29]. The increased expression of CD163 is associated with a poor overall survival in various cancers [30,31]. In line with this, CD163 has been suggested as a novel therapeutic target for glioma [31]. Interestingly, our data displayed that glioma induced CD163 expression on monocytes can be prevented by reducing the sialic acid content of glioma cells using a sialic acid inhibitor. Moreover, the sialic acid-dependent regulation of CD163 expression was observed both in healthy control and glioma patient-derived monocytes.

Tumor-associated myeloid cells can negatively affect tumor immunity through secretion of cytokines. However, cytokines have been recognized for their dual role in anti-tumor immunity. For instance, IL-6 can induce tumor-associated macrophages supporting tumor development, but IL-6 has also been associated with upregulation of CD40 on these macrophages [32]. CD40 is required for myeloid activation, and is therefore needed for myeloid-mediated tumor control. Upon co-culture with T98G cells reduced in sialic acid content, monocytes displayed an enhanced potential to secrete both TNFα and IL-6 upon LPS stimulation. To validate the involvement of the Siglec–sialic acid axis in this response, Siglec receptors on monocytes were activated using monoclonal antibodies in combination with LPS stimulation. This resulted in diminished TNFα secretion, but no differences in IL-6 were observed. While the T98G-monocyte co-culture experiments lasted 4 days, the Siglec triggering experiments lasted 24 h. The kinetics of IL-6 could explain the discrepancy in IL-6 outcome between the two experiments. Additionally, T98G cells harbor more inflammatory cues besides sialic acids that could be interfered by the Siglec–sialic acid axis but would be missed with solely Siglec triggering. 

Together, our data suggest that sialic acids on glioma cells are at least partly responsible for the polarization of primary human myeloid cells into a tumor-supportive phenotype. This aligns with the recent findings by Schmassmann et al. [17], where disruption of the Siglec–sialic axis promoted the anti-tumor immune response in murine glioblastoma models. Furthermore, we have shown that monocytes isolated from glioma patients are still capable to repolarize into a phenotype contributing to tumor control comparable to healthy control monocytes. The data could be strengthened by looking at freshly isolated glioma cells and tumor-infiltrating MDSCs, as well as by investigating alternative mechanisms through which glioma cells support the immune-suppressive functions of MDSCs. Furthermore, future studies focusing blocking Siglec–sialic acid interactions are required to further investigate the therapeutic potential of these interactions for glioma patients.

## Data Availability

Data is contained within the article.

## Supplementary Materials

The following supporting information can be downloaded at: https://www.mdpi.com/article/10.3390/pharmaceutics16070953/s1, Figure S1. The sialylation status of the T98G cells at the end of the monocyte co-culture experiment. T98G cells were treated with the sialic acid inhibitor SiaFEt. After 4 days, the T98G cells were extensively washed and added to the monocytes for the co-culture experiments. After another 4 days, the expression of α2-3 or α2-6-linked sialic acid and the underlying glycan structure of sialic acid (galactose) on T98G cells was analyzed with MALII, SNA and PNA lectins respectively. T98G cells treated with SiaFEt displayed a sialic acid expression reduction and an increased PNA binding at day 4 of the T98G-monocyte co-culture.
