# Supplementary material for: Glioma-Associated Sialoglycans Drive the Immune Suppressive Phenotype and Function of Myeloid Cells
Source: Pharmaceutics. 2024 Jul 19;16(7):953. doi: 10.3390/pharmaceutics16070953 (PMC11279593; doi:10.3390/pharmaceutics16070953)
Supplement: Supplementary file 1 [file pharmaceutics-16-00953-s001.zip › pharmaceutics-3067543-supplementary.pdf]

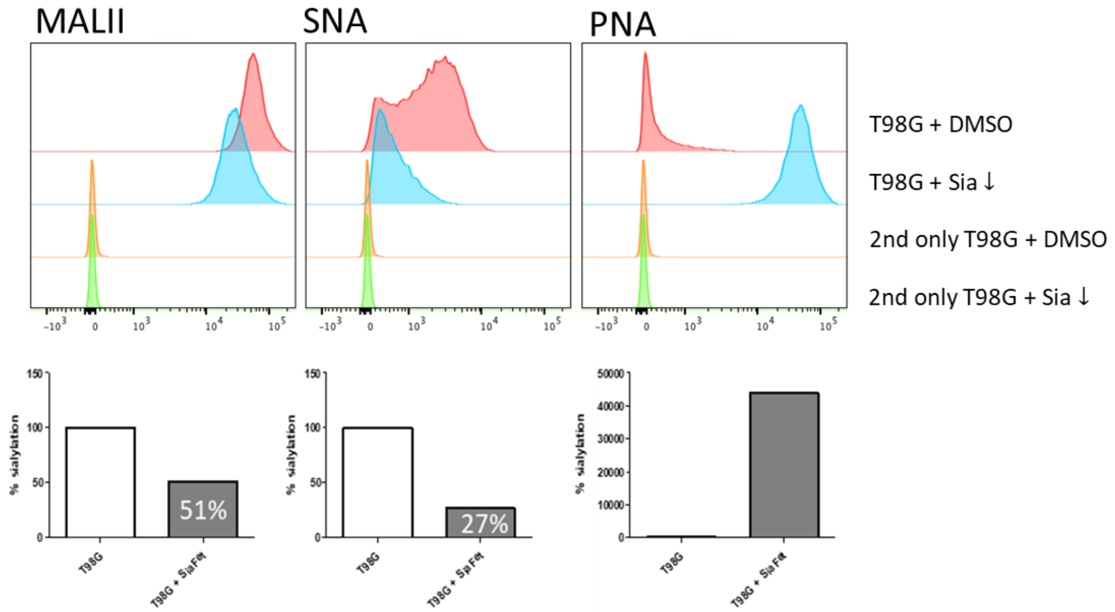

Supplementary Figure S1. The sialylation status of the T98G cells at the end of the monocyte co-culture experiment. T98G cells were treated with the sialic acid inhibitor SiaFEt. After 4 days, the T98G cells were extensively washed and added to the monocytes for the co-culture experiments. After another 4 days, the expression of α2-3 or α2-6-linked sialic acid and the underlying glycan structure of sialic acid (galactose) on T98G cells was analyzed with MALII, SNA and PNA lectins respectively. T98G cells treated with SiaFEt displayed a sialic acid expression reduction and an increased PNA binding at day 4 of the T98G-monocyte co-culture.
